# Association of a Crime Prevention Program for Boys With Mortality 72 Years After the Intervention: Follow-up of a Randomized Clinical Trial

**DOI:** 10.1001/jamanetworkopen.2019.0782

**Published:** 2019-03-29

**Authors:** Brandon C. Welsh, Steven N. Zane, Gregory M. Zimmerman, Alexis Yohros

**Affiliations:** 1 School of Criminology and Criminal Justice, Northeastern University, Boston, Massachusetts; 2 College of Criminology and Criminal Justice, Florida State University, Tallahassee

## Abstract

**Importance:**

Mortality is an important outcome in evaluating crime prevention programs, but little is known about the effects on mortality during the full life course.

**Objective:**

To determine the long-term outcomes of a crime prevention program on mortality and whether the iatrogenic effects on mortality observed in middle age persist or change in old age.

**Design:**

This longitudinal follow-up was conducted in a cohort of boys included in a matched-pair randomized clinical trial (the Cambridge-Somerville Youth Study). Six hundred fifty boys aged 5 to 13 years who lived and attended public and parochial schools in working-class areas of Cambridge and Somerville, Massachusetts, and were identified as at risk for antisocial behavior were matched by age and demographic characteristics. One member of each pair was randomly allocated to the treatment condition. The original trial was performed from June 1, 1939, to December 1945. Follow-up in the present analysis was performed from January 2016 through June 2018.

**Interventions:**

Treatment group participants received individual counseling through a range of activities and home visits for an extended duration (mean, 5.5 years). Control group participants received no special services.

**Main Outcomes and Measures:**

The 4 outcomes of interest include age at mortality, mortality at latest follow-up, premature mortality (younger than 40 years), and cause of mortality (natural vs unnatural).

**Results:**

In the original analysis, 650 participants were matched and randomized to treatment or control conditions, of whom 506 were retained in the analysis (mean [SD] age, 9.8 [1.7] years). Through early 2018, records for 488 participants (96.4%) were located. A total of 446 participants were confirmed dead (88.1%) and 42 alive (8.3%). Matched-pairs analyses showed no significant differences between the treatment and control groups for mortality at latest follow-up (relative risk [RR], 1.05; 95% CI, 0.99-1.11), premature mortality (RR, 1.15; 95% CI, 0.55-2.43), or cause of mortality (RR, 1.19; 95% CI, 0.65-2.18) (*P* > .05 for all). Cox proportional hazard regression indicated no difference in time to death between groups (hazard ratio, 1.18; 95% CI, 0.98-1.41; *P* = .09).

**Conclusions and Relevance:**

Iatrogenic effects on mortality were not detected in this long-term follow-up. The longitudinal analysis provides information on the utility of life-long assessments of crime prevention programs and draws attention to the need for quality-of-life assessments of participants and their children.

## Key Points

**Question:**

What are the long-term effects of a crime prevention program on mortality?

**Findings:**

In this longitudinal follow-up of a randomized clinical trial of 506 boys who received individual counseling through a range of activities and home visits for an extended duration vs no special services, no important differences were found between treatment and control group participants for mortality outcomes 72 years after the intervention, suggesting that earlier observed iatrogenic effects did not persist.

**Meaning:**

Continued research on studies with iatrogenic effects is needed to better understand why and when some programs cause harm and to help mitigate their perpetuation.

## Teaser

This longitudinal follow-up of a randomized clinical trial assesses the long-term association of counseling and a crime prevention program with mortality among boys at risk for delinquency.

## Introduction

Antisocial, criminal, and violent behavior is highly associated with health problems and premature mortality during the life course.^1,2,3,4^ During the past 2 decades, focus on early prevention of criminal and violent behavior has increased,^5,6,7^ producing an emerging evidence base on promising and effective modalities.^5,8,9,10,11^

The first randomized clinical trial to investigate effects of a preventive intervention on children’s criminal behavior, the Cambridge-Somerville Youth Study (CSYS), was initiated in the 1930s^12,13,14^ by Richard Clarke Cabot, MD, a physician and professor of clinical medicine and social ethics at Harvard University.^15,16^ The preventive intervention holds some similarities to mentoring programs today.^17^

The CSYS has been the subject of 3 prior assessments of criminal behavior and other outcomes in 1948, 1956, and 1975 through 1979. Results from the first 2 assessments did not show any effects on criminal behavior.^18,19,20^ The later assessment,^21,22^ performed 30 years after the intervention (mean [SD] age of participants, 47.4 [1.7] years), found that the program produced iatrogenic effects. Compared with the control group, treatment group participants were significantly more likely to commit 2 or more crimes, have symptoms of alcoholism, manifest signs of mental illness, have occupations with lower prestige, have high blood pressure or heart trouble, and experience premature mortality (younger than 35 years).^21,22^

These results were of great concern to practitioners of the day. Individual counseling for at-risk children and youth, albeit with more structure, was becoming a prominent approach in social work.^23^ For researchers and policy makers, the results reaffirmed that social programs with their good intentions do not guarantee positive effects and in fact carry the potential to cause harm.^24^ The study’s results also drew attention to the need for long-term follow-ups of preventive interventions for the benefit of science and public policy.^25^

We investigated whether the CSYS program’s iatrogenic effects on mortality observed in middle age have persisted or changed in old age, in what is to our knowledge the longest assessment of a preventive intervention. Beyond the utility of long-term assessments of health interventions in general,^26,27^ we were motivated by the strong association of criminal offending and mortality, as well as the paucity of research on the effects of crime prevention programs on mortality.^28,29,30^ Moreover, this knowledge can be greatly improved by following up individuals into old age. Key reasons include the availability of a more complete set of death records and the increased opportunity to examine natural causes of mortality.^1,31^

## Methods

### Study Design, Setting, and Participants

We conducted a longitudinal follow-up of a cohort of boys included in a matched randomized clinical trial (the CSYS). The original trial ran from June 1939 through December 1945. The present longitudinal analysis was performed from January 2016 through June 2018 with the approval of the institutional review board of Northeastern University, Boston, Massachusetts. This follow-up study followed the Strengthening the Reporting of Observational Studies in Epidemiology (STROBE) reporting guidelines.^32^ Written informed consent was obtained from all participants as part of the last follow-up.

The identification and recruitment of boys for the CSYS was performed by a selection committee created by the director and consisting of 3 prominent practitioners in juvenile and criminal justice.^33^ Beginning in 1935, the committee’s charge was to recruit boys who were aged 5 to 13 years, lived and attended public and parochial schools in working-class areas of Cambridge and Somerville, Massachusetts, and were considered predelinquent. Characteristics of predelinquency included “persistent truancy, persistent breaking of the rules, sex difficulties, petty pilfering and stealing, failing to return home after school, and, among the kindergartners, temper tantrums.”^34^ To avoid labeling effects, the study included a similar number of boys considered average and difficult. Boys were the exclusive focus of the study because of the prevailing view at the time that only males engaged in criminal behavior.

The committee identified a large proportion of boys from referrals, mostly by local schools (approximately 77%), as well as by local welfare agencies, churches, and the police. Information on the boys was collected from a wide range of sources, including elementary school teachers, juvenile courts, physicians, and the parents of eligible boys. After the exclusion of hundreds of boys (detailed in the Results section), case files of eligible boys were turned over to a group of psychologists employed by the study to perform matching.

Staff psychologists first observed a group of older boys (n = 80) on overnight camping trips to determine relevant social and personality traits for the purpose of operationalizing matching parameters. They then matched all boys (n = 650) on 142 variables (rated on an 11-point scale) covering a wide range of characteristics, including physical health, emotional and social adjustment, father’s occupation, teacher ratings of average or difficult, mental health, aggressiveness, acceptance of authority, discipline, and delinquency or disruption in the home environment.^18^ Information on these variables was drawn from multiple sources, including interviews with the boys and their parents, teacher reports, and police records. This process resulted in 325 matched pairs.

One member of each matched pair was chosen at random (on a coin toss) to be in the treatment group. Overseen by the study director, the process of random allocation was staggered, beginning on November 1, 1937, and ending on May 13, 1939, with the intervention officially starting on June 1, 1939. Two violations of the random allocation procedure occurred. First, 8 boys were matched after the program began. Second, “brothers were assigned to that group to which the first of siblings was randomly assigned.”^35^^(p199)^ This violation involved a total of 40 boys, 21 in the treatment group and 19 in the control group.^35^ Likely due to the study period, there is no indication that power calculation influenced this sample size determination.

### Study Treatment

In the 1930s, the preventive intervention was described as character development through positive role models, also termed *directed friendship*.^36^ Boys in the treatment group received individual counseling and home visits by paid professional counselors, better known as case workers at the time. Strengthening the family unit was key to Cabot’s vision for directed friendship. The boys were paired with counselors who sought to provide positive influences in their lives and “supplement but not replace what would normally be a satisfactory parent-child relationship.”^37^^(p143)^ Counseling activities included taking the boys on trips and to recreational activities, tutoring them in reading and arithmetic, encouraging them to participate in the YMCA and summer camps, playing games with them at the project’s center, encouraging them to attend church, and giving advice and general support to the boys’ families. Participants were enrolled in the program for a mean of 5.5 years, with case workers visiting the boys in the treatment group a mean of 2 times per month. The control group received no special services.

### Data Collection

After completion of the program, 4 assessments covered major lifetime periods, including transition from adolescence to early adulthood (1948), early adulthood (1956), middle-age (1975-1979), and in the present study, old age (2016-2018). Data were collected for the boys and their parents while the program was in operation; the boys were the sole focus of the subsequent assessments.

Tracing participants for the latest assessment began with a search for and collection of official death records. A comprehensive and systematic set of procedures was used, building on those procedures used in the last follow-up and by others.^38^ We used the Massachusetts Registry of Vital Records and Statistics (MA Registry) and the National Death Index (NDI) of the National Center for Health Statistics, a centralized database of death record information for all US states and territories, as data sources.

We made physical visits to the MA Registry during 2016 and 2017 and searched electronic records through the end of 2016. Date and cause of death were coded, and all information was verified with paper copies of death certificates. The next step involved contracting with the NDI to search for death records of the remaining participants. The NDI includes deaths since 1979 and was updated on an annual basis with a lag time of approximately 12 months. Searches of death records were performed by the NDI staff through the end of 2017. A service called NDI Plus, which provides information on cause of death, was also used.

The search for the other participants involved harnessing digital resources that are now widely available to the public.^39^ This search began with Ancestry.com (https://www.ancestry.com/), a subscription-based genealogy website that draws on various national and local sources, including records for birth, marriage, and death (official and unofficial). Searches on the site first involved looking up birth, marriage, and census records to cross-reference spellings of the names and dates of birth.

Ancestry.com was also used to identify any additional deaths. National and local burial, cemetery, and obituary collections were of particular interest. Local and national obituary sites that provided even more up-to-date information were also searched. These sites included Legacy.com (https://www.legacy.com/), Bostonglobe.com (https://www.bostonglobe.com/), and Findagrave.com (https://www.findagrave.com/). Obituaries of parents, siblings, and spouses also provided indications of whether a participant may have been dead or alive at the time of death of their relative. Participants were considered reliable matches if official death records or unofficial death announcements matched in full name, date of birth, and birthplace in Massachusetts. A return visit to the MA Registry (in June 2018) was used to verify death records and collect information on cause of death for participants identified as deceased from other sources.

To further ascertain whether participants were alive, we searched FamilyTreeNow.com (https://www.familytreenow.com/), with a particular focus on its Living People Records. This site consists of public records updated weekly from numerous sources, such as telephone directories, property records, birth and death records, magazine subscriptions, and voter registration. Participants were judged to be alive if the site records matched the participant in full name, date of birth, and place of birth, and if no official or unofficial death records were found in any other sources.

### Outcomes

We had 4 outcomes of interest: mortality at latest follow-up, age at mortality, premature mortality, and cause of mortality. Mortality is measured as a binary outcome to indicate whether the participant died by December 2017. As with the most similar long-term follow-up of mortality outcomes,^1^ premature mortality is defined here as death at younger than 40 years, and a series of binary variables for mortality by 5-year cutoffs were created to explore different ages for premature mortality (described in the Results section). Cause of mortality was coded as natural (including cardiovascular diseases, cancers, and other natural diseases) or unnatural (including substance or alcohol abuse, accidents, homicides, suicides, and infections). Among those who died (n = 446), age at mortality was measured as a continuous variable.

### Statistical Analysis

The primary phase of the analysis involved cohort matched-pairs analyses. For categorical data collected on matched pairs, the McNemar test^40^ provided the conventional analysis of marginal homogeneity, testing the null hypothesis that the probabilities of each death-related outcome are the same for each member of the pair.^41^ The McNemar test, which is generalized beyond pairs in the Mantel-Haenszel test,^42^ uses a 2 × 2 contingency table for matched cohort data, when the treatment variable and outcome variable are dichotomous. The Stata 15 plugin *csmatch* uses the more general Mantel-Haenszel test for variance estimation.^43^ This method computes the relative risk (RR; rather than odds ratios reported from the McNemar test) for the outcome between the paired groups, which is “exactly the same as the relative risk which can be derived by stratifying the analysis on the matched pairs and using Mantel-Haenszel methods to summarize the data.”^44^^(p132)^ Using this approach, we conducted matched-pairs analyses for 3 binary outcomes of interest: mortality by end of follow-up, premature mortality (younger than 40 years), and cause of mortality. Differences in age at mortality could not be examined using matched-pairs analysis (because the outcome is continuous), so we also conducted survival analysis. Time to death for the 2 groups was examined using Kaplan-Meier methods and compared using a Cox proportional hazards regression model. All tests used 2-sided hypothesis testing with an α of .05. All tests were performed using Stata software (version 15; StataCorp).

## Results

### Study Participants and Descriptive Characteristics

**Figure 1.**
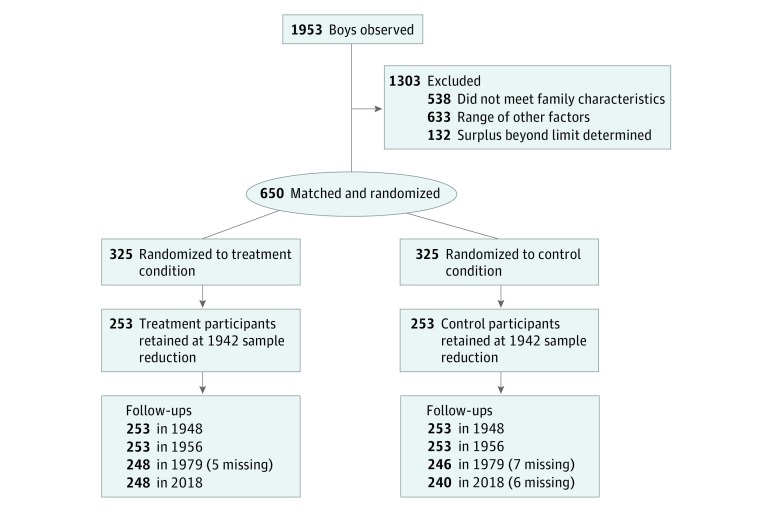
CONSORT Flow Diagram The sample reduction in 1942 was owing to resource shortages (eg, rationing of gas) because of the United States’ involvement in World War II.

The initial sample of the CSYS consisted of 650 boys or 325 matched pairs (Figure 1). A total of 1953 boys referred to the study underwent initial screening, from whom 782 were selected for consideration for inclusion in the study. Of the 1171 boys who were excluded, 538 did not meet the family characteristics (eg, low income) and 633 included a range of factors, such as being too old, moved out of town, or could not be located (more than one-half); insufficient information “upon which to base a judgment”^45^^(p52)^ (at least one-third); and miscellaneous reasons, including death, lack of cooperation, and mostly duplication of names.^45^ The additional 132 boys who were not included represented a “surplus … beyond the limit determined—325 for the treatment group and 325 for the control group.”^45^^(p54)^

In 1942, the sample was scaled back to 506 participants (or 253 matched pairs). This reduction was a result of resource shortages (eg, rationing of gas) because of the United States’ involvement in World War II (this situation also caused the program to end in 1945). According to McCord,^46^^(p523)^ “When a boy was dropped from the treatment program, his matched mate was dropped from the control group.” A comparison of the 253 remaining pairs indicated no statistically significant differences between the 2 groups on a wide range of variables (eg, age, IQ, referral to the study as average or difficult, mental health). Several criteria were used by the developers to reduce the study’s sample, including boys’ cooperativeness with the counselor, extent of the effort already spent by the counselors, and travel distance (for families who had moved too far away). This final population served as the sample for all assessments of the program. The age range at intervention implementation was 5 to 13 years (mean [SD], 9.8 [1.7] years) (Table 1). The racial composition of the study population included 462 white (91.3%), 41 African American (8.1%), and 3 mixed-race (0.6%) participants.

**Table 1.** Baseline Characteristics of Study Boys and Their Parents

Characteristic	No. (%) by Study Group^a^	
	Treatment (n = 253)	Control (n = 253)
Age of target boy, mean (SD), y	9.8 (1.6)	9.8 (1.7)
Race/ethnicity of target boy		
White	231 (91.3)	231 (91.3)
Black or African American	22 (8.7)	19 (7.5)
Mixed	0	3 (1.2)
Neighborhood		
Good to fair	78 (33.5)	96 (38.4)
Poor to worst	155 (66.5)	154 (61.6)
Presence of parents		
Both present	141 (55.7)	158 (62.5)
Father absent	78 (30.8)	58 (22.9)
Mother absent	15 (5.9)	15 (5.9)
Both absent	19 (7.5)	22 (8.7)
Father’s educational attainment		
None to little	185 (85.3)	179 (88.2)
High school graduate	17 (7.8)	15 (7.4)
Postsecondary	15 (6.9)	9 (4.4)
Father’s employment		
Regular	105 (43.4)	106 (46.1)
Irregular	118 (48.8)	81 (35.2)
Unemployed	19 (7.9)	43 (18.7)
Father’s occupation		
Professional or white collar	24 (9.8)	36 (15.2)
Skilled	76 (31.0)	70 (29.5)
Unskilled	145 (59.2)	131 (55.3)
Mother’s educational attainment		
None to little	191 (88.0)	189 (90.4)
High school graduate	18 (8.3)	14 (6.7)
Postsecondary	8 (3.7)	6 (2.9)
Mother’s employment outside home		
Regular	35 (14.0)	25 (10.0)
Irregular	52 (20.8)	35 (14.0)
None	163 (65.2)	190 (76.0)

^a^ Owing to missing data, totals for each characteristic may not sum the numbers in the column headings.

We identified 446 participants as deceased (88.1%), 42 as living (8.3%), and 18 as missing (3.6%). For the treatment group, 231 were confirmed deceased (91.3%), 17 alive (6.7%), and 5 missing (2.0%); for the control group, 215 were confirmed deceased (85.0%), 25 alive (9.9%), and 13 missing (5.1%). Twelve of the missing participants were not located in the 1979 follow-up (97.6% sample retention) and an additional 6 participants were identified as missing in the present follow-up, for a total sample retention of 488 (96.4%). The 18 missing participants were part of 18 matched pairs, and all were dropped, for a final sample of 235 matched pairs (92.9%).

### Outcomes

For mortality at latest follow-up, as noted above, 446 participants were confirmed deceased (88.1%). We identified 32 participants who died at younger than 40 years (6.3%), coded as premature mortality. Among total deceased, the median age of mortality was 69.5 years (interquartile range, 56-79 years). Cause of mortality was missing for an additional 14 participants, and mortality for the remaining participants was coded using the following categories: cardiovascular diseases (n = 144), cancers (n = 118), other natural diseases (n = 127), substance or alcohol abuse (n = 10), accidents (n = 18), homicide (n = 2), suicide (n = 7), and infections (n = 6). Unnatural causes accounted for 43 deaths (10.0%). Table 2 reports outcomes for treatment and control groups.

**Table 2.** Mortality Outcomes by Study Group

Outcome	Treatment Group (n = 253)	Control Group (n = 253)
Categorical, No. (%)		
Mortality at latest follow-up	231 (91.3)	215 (85.0)
Premature mortality (younger than 40 y)	18 (7.1)	14 (5.5)
Unnatural cause of mortality	25 (9.9)	18 (7.1)
Continuous, median (IQR)		
Age at death	69 (55-77)	70 (56-80)

Abbreviation: IQR, interquartile range.

### Main Results

Results of the cohort matched-pairs analyses are reported in Table 3. For mortality at latest follow-up, of the 235 matched pairs, 197 pairs were concordant with both members deceased, 2 were concordant with both members alive, 23 were discordant with only the treatment group participant deceased, and 13 were discordant with only the control deceased (eTable 1 in the Supplement). The RR of mortality, expressed as the ratio of the rates of death in the treatment group (220 of 235) to those of the control group (210 of 235), was not significant (RR, 1.05; 95% CI, 0.99-1.11).

**Table 3.** Relative Risk of Mortality Outcomes, by Matched Pairs^a^

Outcomes	RR (95% CI)
Mortality at latest follow-up	1.05 (0.99-1.11)
Premature mortality (younger than 40 y)	1.15 (0.55-2.43)
Unnatural cause of mortality^b^	1.19 (0.65-2.18)

Abbreviation: RR, relative risk.

^a^ Includes 235 matched pairs.

^b^ Includes 186 matched pairs.

For premature mortality (younger than 40 years), of the 235 matched pairs, 0 pairs were concordant with both members deceased, 207 were concordant with both members alive, 15 were discordant with only the treatment group participant dead, and 13 were discordant with only the control dead (eTable 2 in the Supplement). The RR of premature mortality was not significant (1.15; 95% CI, 0.55-2.43). Further, the RR of mortality between groups did not reach significance for any period of premature mortality from younger than 40 to younger than 65 years (in 5-year cutoffs).

**Figure 2.**
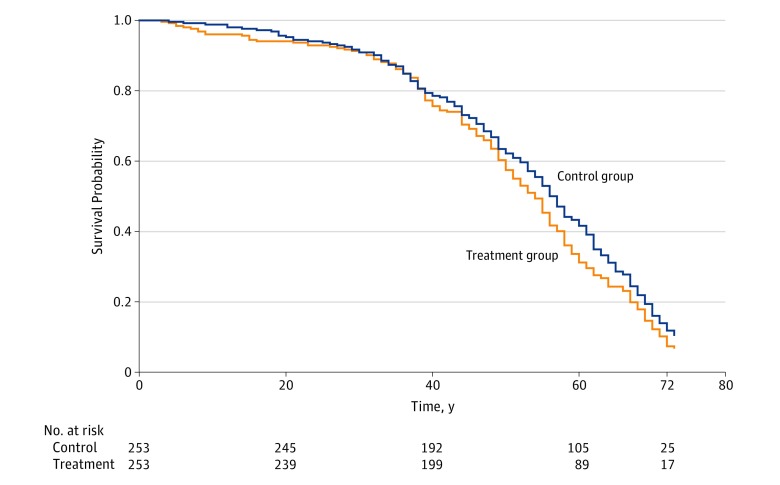
Kaplan-Meier Survival Estimates for Treatment and Control Group Participants Data were obtained from the end of the trial (December 1945) through the present longitudinal analysis from January 2016 through June 2018.

For cause of mortality, of the 186 matched pairs with both members dead at the latest follow-up, 3 pairs were concordant with both members dead due to unnatural causes, 154 were concordant with both members dead due to natural causes, 16 were discordant with only the treatment group participant dead due to unnatural causes, and 13 were discordant with only the control dead due to unnatural causes (eTable 3 in the Supplement). The RR of unnatural death was not significant (1.19; 95% CI, 0.65-2.18). Last, for age at mortality, Cox proportional hazard regression indicated no difference in time to death between groups, with a nonsignificant hazard ratio for the treatment group vs control group of 1.18 (95% CI, 0.98-1.41; *P* = .09) (Figure 2).

## Discussion

With the present long-term assessment of the first randomized clinical trial to prevent criminal behavior, we set out to investigate whether the iatrogenic effects on mortality observed in middle age^21,22^ have persisted or changed in old age. We observed no statistically significant differences between men in the treatment and control groups for the outcomes of interest, including mortality at current follow-up, premature mortality, cause of mortality, and age at mortality. In other words, the iatrogenic effects on mortality observed in middle age were not observed in old age.

The results highlight the importance of long-term assessments of preventive interventions that aim to change the life course of individuals who are at high risk for serious social, mental, and physical health problems during their lifetimes.^47^ For the past 40 years,^21^ the CSYS was viewed as an example of a preventive intervention that had serious iatrogenic effects and caused great concern.^48,49^ Following these unexpected iatrogenic effects, the primary message of McCord^23^ was the importance of using experimental designs to test all preventive interventions. We believe that the present results reinforce the convictions of Cabot^37^ and McCord^23^ that randomized clinical trials are needed for all preventive interventions, including those meant to prevent social problems.

In the end, we cannot say definitely why the iatrogenic effects observed in middle age were not observed in old age. The observed iatrogenic effects on mortality in middle age may have been a singular event, irrespective of their concordance with effects for a wide range of other outcomes (eg, criminal offending, mental health). Other preventive interventions have reported short-term iatrogenic effects,^24,50^ but to our knowledge no studies have reported short-term iatrogenic effects on mortality.

### Limitations

Several limitations confront the CSYS and the current assessment. As discussed above, 2 violations of the random allocation protocol involved a total of 48 participants (7.4%) from the initial sample of 650 boys. In addition, the trial’s initial sample was reduced in 1942 to 253 matched pairs. Although the sample reduction was disappointing for overall power, the dropping of matched pairs addresses concerns of differential attrition.

The generalizability of the present study’s effects may be somewhat limited for other programs. Questions can also be raised about its relevance to preventive interventions today; for example, its focus on boys only and those who are mostly white. Our study was not able to assess the program’s effects on criminal offending (the other main outcome that can be investigated using administrative records) into old age.

## Conclusions

Lifelong follow-ups of crime prevention programs and health interventions in general are needed to assess the full effects on the lives of participants and capture continuity or discontinuity of important outcomes during the life course. From a historical perspective, the CSYS has had a major influence on other crime prevention trials that have shown long-term positive effects.^51,52^ From a contemporary perspective, the present life-span assessment calls attention to the need for continued research on iatrogenic effects, with the aim to better understand why and when some programs cause harm and to help mitigate their perpetuation.^23,53,54^ We believe that the next step with this study should be to investigate its positive and/or negative effects on different aspects of the quality of life of the participants and their own children. Our aim is now to further this long-term follow-up and analyze the details of the lives of the CSYS participants and of their children to clarify this all-important issue.

## Notes

Supplement. **eTable 1.** Mortality at Latest Follow-up, by Matched Pairs (n = 235) **eTable 2.** Premature Mortality, by Matched Pairs (n = 235) **eTable 3.** Cause of Mortality, by Matched Pairs (n = 186)
