# Supplementary material for: Association of a Crime Prevention Program for Boys With Mortality 72 Years After the Intervention: Follow-up of a Randomized Clinical Trial
Source: JAMA Netw Open. 2019 Mar 29;2(3):e190782. doi: 10.1001/jamanetworkopen.2019.0782 (PMC6450308; doi:10.1001/jamanetworkopen.2019.0782)
Supplement: Supplement. — eTable 1. Mortality at Latest Follow-up, by Matched Pairs (n = 235) eTable 2. Premature Mortality, by Matched Pairs (n = 235) eTable 3. Cause of Mortality, by Matched Pairs (n = 186) [file jamanetwopen-2-e190782-s001.pdf]

Supplementary Online Content

Welsh BC, Zane SN, Zimmerman GM, Yohros A. Association of a crime prevention program for boys with mortality 72 years after the intervention: follow-up of a randomized clinical trial. *JAMA Netw Open*. 2019;2(3):e190782. doi:10.1001/jamanetworkopen.2019.0782

eTable 1. Mortality at Latest Follow-up, by Matched Pairs (n = 235)

eTable 2. Premature Mortality, by Matched Pairs (n = 235)

eTable 3. Cause of Mortality, by Matched Pairs (n = 186)

This supplementary material has been provided by the authors to give readers additional information about their work.

© 2019 Welsh BC et al. *JAMA Network Open.*

eTable 1. Mortality at Latest Follow-up, by Matched Pairs (n = 235)

**Treatment**	**Control**		
	Deceased	Alive	Total
Deceased	197	23	220
Alive	13	2	15
Total	210	25	235

© 2019 Welsh BC et al. *JAMA Network Open.*

eTable 2. Premature Mortality, by Matched Pairs (n = 235)[a]

**Treatment**	**Control**		
	Deceased	Alive	Total
Deceased	0	15	15
Alive	13	207	220
Total	13	222	235

[a]Indicates younger than 40 years.

© 2019 Welsh BC et al. *JAMA Network Open.*

eTable 3. Cause of Mortality, by Matched Pairs (n = 186)

**Treatment**	**Control**		
	Unnatural	Natural	Total
Unnatural	3	16	19
Natural	13	154	167
Total	16	170	186

© 2019 Welsh BC et al. *JAMA Network Open.*
